# Large Language Model Approach for Zero-Shot Information Extraction and Clustering of Japanese Radiology Reports: Algorithm Development and Validation

**DOI:** 10.2196/57275

**Published:** 2025-01-23

**Authors:** Yosuke Yamagishi, Yuta Nakamura, Shouhei Hanaoka, Osamu Abe

**Affiliations:** 1 Division of Radiology and Biomedical Engineering, Graduate School of Medicine, The University of Tokyo, Tokyo, Japan; 2 Department of Computational Diagnostic Radiology and Preventive Medicine, The University of Tokyo Hospital, Tokyo, Japan

**Keywords:** radiology reports, clustering, large language model, natural language processing, information extraction, lung cancer, machine learning

## Abstract

**Background:**

The application of natural language processing in medicine has increased significantly, including tasks such as information extraction and classification. Natural language processing plays a crucial role in structuring free-form radiology reports, facilitating the interpretation of textual content, and enhancing data utility through clustering techniques. Clustering allows for the identification of similar lesions and disease patterns across a broad dataset, making it useful for aggregating information and discovering new insights in medical imaging. However, most publicly available medical datasets are in English, with limited resources in other languages. This scarcity poses a challenge for development of models geared toward non-English downstream tasks.

**Objective:**

This study aimed to develop and evaluate an algorithm that uses large language models (LLMs) to extract information from Japanese lung cancer radiology reports and perform clustering analysis. The effectiveness of this approach was assessed and compared with previous supervised methods.

**Methods:**

This study employed the MedTxt-RR dataset, comprising 135 Japanese radiology reports from 9 radiologists who interpreted the computed tomography images of 15 lung cancer patients obtained from Radiopaedia. Previously used in the NTCIR-16 (NII Testbeds and Community for Information Access Research) shared task for clustering performance competition, this dataset was ideal for comparing the clustering ability of our algorithm with those of previous methods. The dataset was split into 8 cases for development and 7 for testing, respectively. The study’s approach involved using the LLM to extract information pertinent to lung cancer findings and transforming it into numeric features for clustering, using the K-means method. Performance was evaluated using 135 reports for information extraction accuracy and 63 test reports for clustering performance. This study focused on the accuracy of automated systems for extracting tumor size, location, and laterality from clinical reports. The clustering performance was evaluated using normalized mutual information, adjusted mutual information , and the Fowlkes-Mallows index for both the development and test data.

**Results:**

The tumor size was accurately identified in 99 out of 135 reports (73.3%), with errors in 36 reports (26.7%), primarily due to missing or incorrect size information. Tumor location and laterality were identified with greater accuracy in 112 out of 135 reports (83%); however, 23 reports (17%) contained errors mainly due to empty values or incorrect data. Clustering performance of the test data yielded an normalized mutual information of 0.6414, adjusted mutual information of 0.5598, and Fowlkes-Mallows index of 0.5354. The proposed method demonstrated superior performance across all evaluation metrics compared to previous methods.

**Conclusions:**

The unsupervised LLM approach surpassed the existing supervised methods in clustering Japanese radiology reports. These findings suggest that LLMs hold promise for extracting information from radiology reports and integrating it into disease-specific knowledge structures.

## Introduction

Natural language processing (NLP) is vital in medicine as it allows the interpretation of textual content in medical documents. Radiology reports, written as free text by experienced radiologists, contain detailed information about medical imaging findings. While medical images are valuable, text-based analysis offers unique advantages in terms of computational efficiency and the ability to capture expert interpretations and observations of radiologists that may not be immediately apparent from images. Natural language processing can effectively extract this information, enhance its utilization, and provide new insights into medical imaging.

Advances in radiological NLP applications are driven by the availability of large datasets [1]. For example, the MIMIC Chest X-ray (MIMIC-CXR) includes more than 200,000 images, English-language reports, and structured data [2]. Numerous NLP models have been developed to summarize and extract clinical entities [3,4]. However, the availability of these datasets in languages other than English is limited.

To address this challenge, the NTCIR-16 Real-MedNLP shared task focused on clustering Japanese radiology reports by case basis. It is a set of Japanese radiology reports authored by different radiologists for the same case series of lung cancer, and the task was to cluster reports that describe the same medical case together [5]. This benchmark evaluates the detailed understanding of radiology reports, as NLP systems must extract sufficient information to recognize reports by diagnosing the same image without being affected by different writing styles.

Clustering is a powerful analytical tool in medicine and has been successfully applied in various clinical domains. Studies have demonstrated its effectiveness in clustering patients based on their clinical characteristics to guide medical decisions, ranging from cancer aftercare planning to pulmonary embolism risk assessment [6,7]. Semantic grouping has enabled efficient insight discovery in medical documents [8] and revealed specialty-specific sublanguages in clinical narratives [9]. Radiology reports are particularly suited for such analyses, as they provide high-quality annotated data despite their free-form nature, offering a more tractable alternative to direct image analysis.

While the participants in the NTCIR-16 (NII Testbeds and Community for Information Access Research) shared task used deep-learning models, their clustering performance was constrained by limited training data. Since then, large language models (LLMs) trained on extensive text corpora, such as ChatGPT and LLaMA [10,11], have emerged. These LLMs, which are adaptable to new tasks with minimal instructions or examples, have demonstrated high performance in extracting information from medical documents, even under zero-shot conditions [12].

This study aimed to evaluate the ability of LLM to understand real radiological reports through an information extraction task and apply this information to clustering, which is a clinically meaningful task.

## Methods

### Study Design and Reporting Guidelines

This retrospective observational study followed the relevant items of the checklist for Artificial Intelligence in medical imaging (CLAIM) guidelines for methodology reporting [13,14]. Although this study analyzed text rather than images, CLAIM was followed because it is an established guideline for AI-based research in radiology and is deemed appropriate for NLP [15-17].

### Algorithm Overview

**Figure 1.**
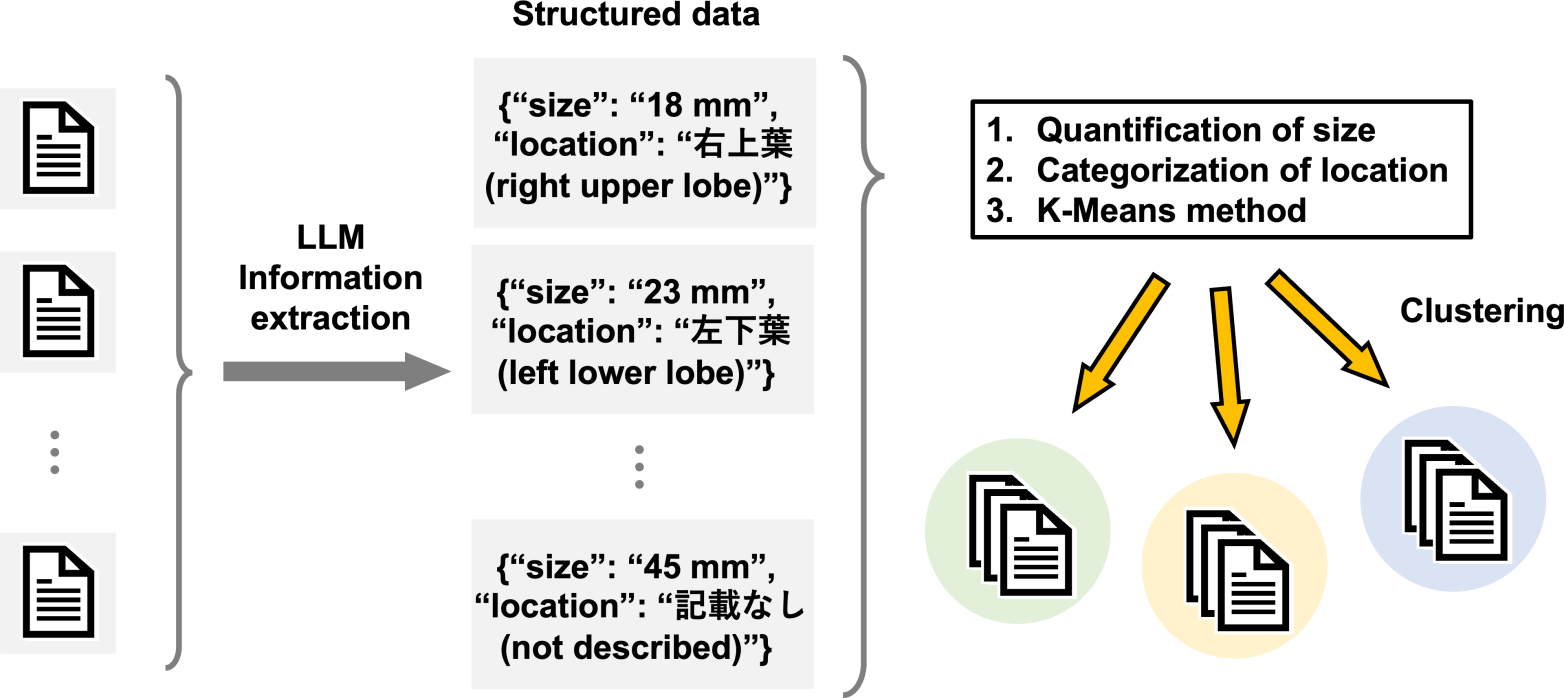
Flowchart of radiology reports clustering using LLM. LLM: large language model.

The proposed algorithm is illustrated in Figure 1. Using the LLM, key lung cancer findings were extracted from radiology reports and quantified to obtain structured data. The structured data were subsequently used for clustering.

### Dataset

**Figure 2.**
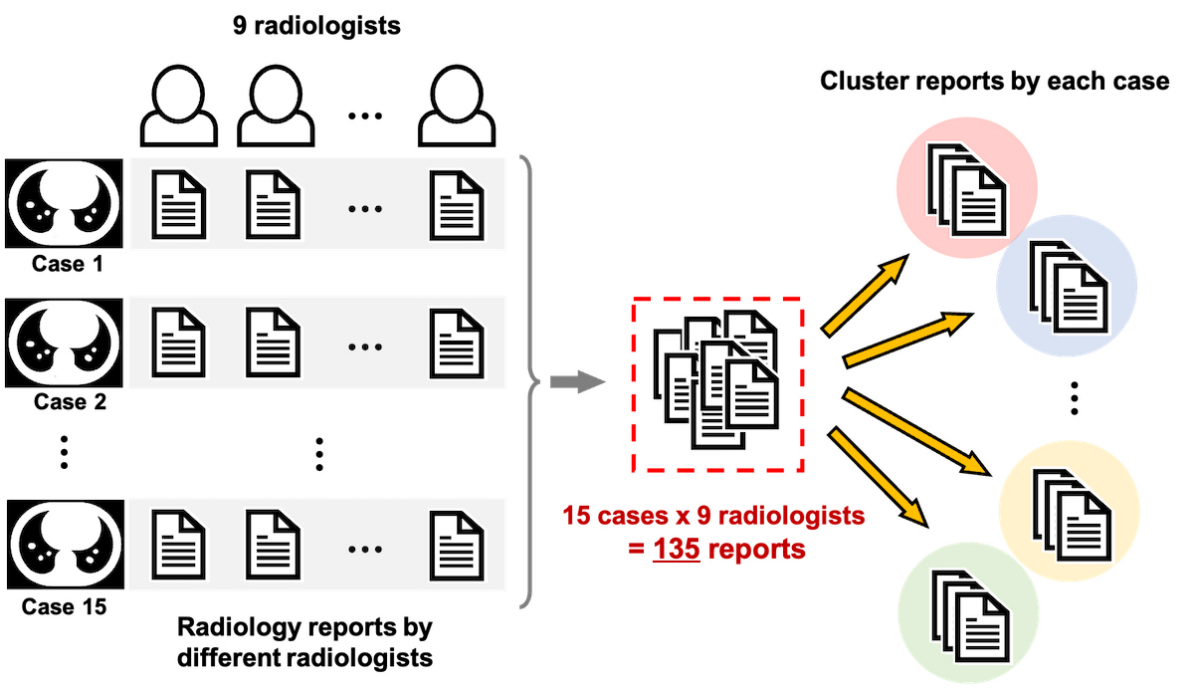
Overview diagram of the radiology report clustering task.

The MedTxt-RR dataset was used in this study [5,18], comprising 135 Japanese radiology reports generated by 9 radiologists who interpreted CT images of 15 lung cancer cases sourced from Radiopaedia [19]. This dataset was used in an NTCIR-16 shared task [5], where participants competed to achieve optimal clustering performance. With each case comprising reports from 9 radiologists, the dataset was suitable for evaluating the clustering performance on a per-case (Figure 2). Eight cases and seven cases were assigned to the development and test sets, respectively. While no model training was conducted using the development set in this study, performance was evaluated on the same data split to facilitate comparison with the shared task results.

### LLM Approach

Radiology reports contain confidential patient information; processing them using a cloud-based LLM, such as ChatGPT, could expose sensitive data externally, raising significant medical safety concerns. Therefore, a publicly available offline model was selected as an alternative approach.

The ELYZA-Japanese-Llama-2-7b-fast-instruct model was employed as the LLM [20]. Adapted from Llama2 and pre-trained using Japanese datasets, this model demonstrated a performance comparable to that of GPT-3.5 on Japanese datasets [21-23].

### Information Extraction

The LLM extracted multiple lung cancer staging parameters from radiology reports, including tumor size, tumor location, and the presence or absence of lymph node enlargement, suggesting metastasis and distant metastasis. To determine the optimal combination of features, clustering performance of the development set were repeatedly measured by using certain features. Consequently, sufficient clustering performance was confirmed achievable using only 3 parameters: tumor size, laterality (left or right), and lung location (upper, hilum, or lower region).

**Figure 3.**
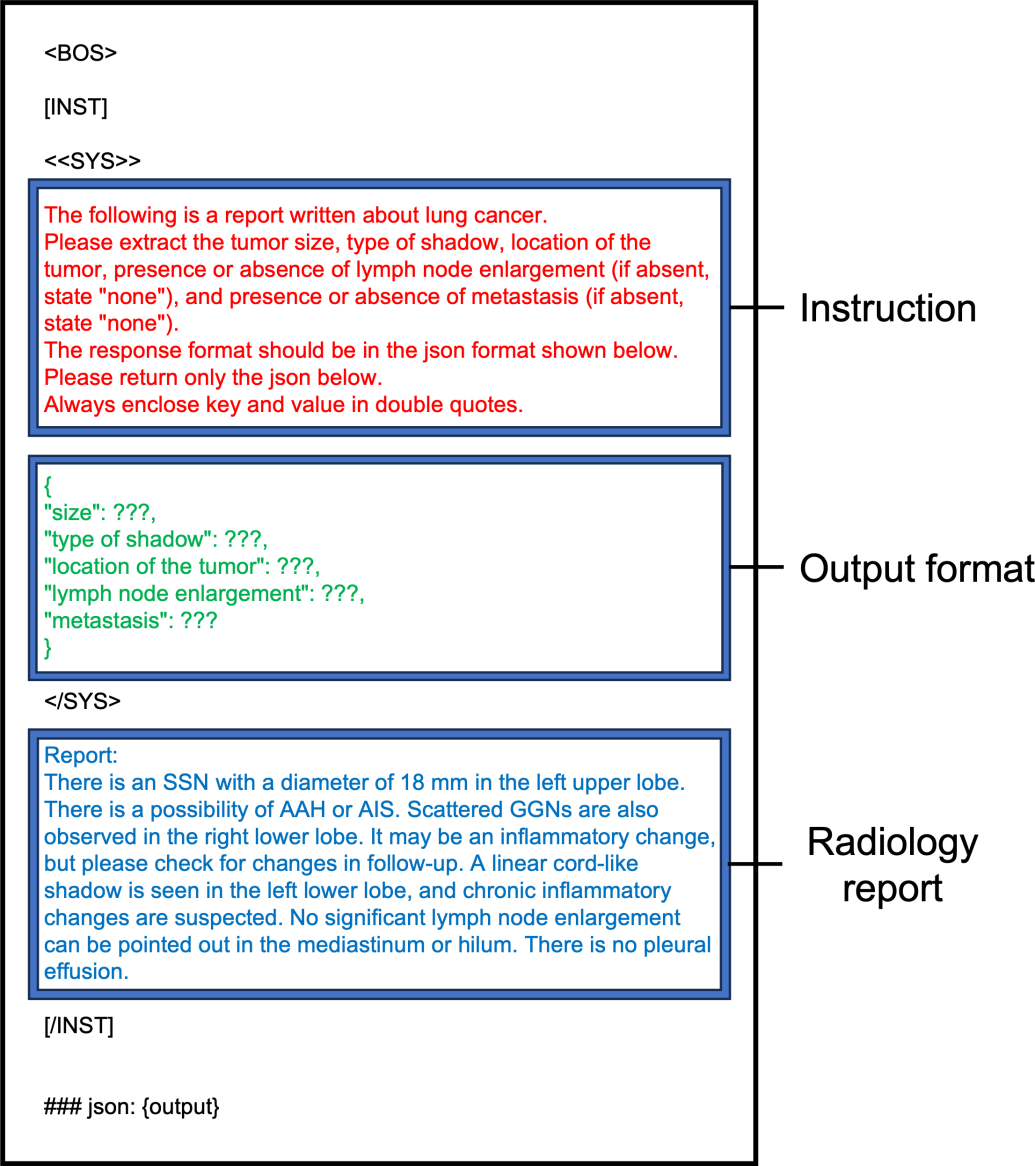
Example of a prompt used as input for the LLM (English translated version). AAH: Atypical Adenomatous Hyperplasia; AIS: Adenocarcinoma in situ; GGN: Ground Glass Nodule; LLM: large language model; SSN: Subsolid Nodule.

The prompt input into the LLM comprises system instructions and output format guidelines using json (JavaScript Object Notation), a standardized text-based format for structured information exchange, where data is organized in key-value pairs, such as {“size”: “45 mm,” “location”: “right upper lobe”}. These system instructions guided the LLM in extracting features from the radiology reports. The details of these prompts are shown in Figure 3 (English version) and Figure S1 in Multimedia Appendix 1 (original Japanese version).

The extracted data were converted into integer vectors comprising the tumor size and other categorical values. Unspecified tumor sizes only described as *large* were replaced with 71 mm, corresponding to the highest category in T classification, where T represents tumor categories in cancer staging. The details of this pipeline can be found in the GitHub Repository [24].

Moreover, a rule-based method was employed as the baseline approach and its performance was compared with that of the proposed method. The rule-based method performs context-sensitive word-based information extraction; the detailed algorithm is shown in Figure S2 in Multimedia Appendix 1.

### Clustering

The resulting numerical matrices were clustered using the K-means algorithm in the scikit-learn library (version 1.3.1). The number of clusters was set to 8, aligning with expected classifications such as disease type or staging, since it was close to the number of test data cases. Centroid initialization used the k-means++ method, with default values for the centroid seed and iteration count, because hyperparameter tuning was not conducted in this zero-shot study.

### Information Extraction Evaluation

Two independent radiologists, a radiology resident with 1-year experience and a board-certified radiologist with 7 years of experience evaluated the accuracy of extracted information. In cases of discrepancy, the final assessment was determined by consensus. Evaluation focused on three key elements: tumor size, location (upper, hilum, or lower), and laterality (left or right). The performance of the LLM-based approach was compared to that of the rule-based method for information extraction. A detailed error analysis was conducted for cases with errors, categorizing them into missing information, false information generation, and extraction of multiple values.

McNemar’s test was performed using Statsmodels (version 0.14.2) [25] to compare performance differences between the LLM-based and rule-based approaches for extracting tumor size and location.

### Clustering Performance Evaluation

We assessed clustering performance using three metrics similar to those used in the shared task [5]: (1) normalized mutual information (NMI) that quantifies the mutual dependence between two clusters, normalized to a 0‐1 scale, with 1 indicating perfect clustering; (2) adjusted mutual information (AMI) which is an adjustment that corrects for NMI, accounting for its tendency to increase with the number of clusters; (3) Fowlkes-Mallows index (FM) that measures the similarity between two clusters by calculating the geometric mean of precision and recall, providing a balanced assessment of clustering accuracy.

### Ethics Consideration

This study involved analysis of human subject data from publicly available radiology reports. All data were completely de-identified and accessible through MedTxt-RR [26]. In accordance with our institution’s policy on research ethics, studies using exclusively de-identified, public datasets are exempt from institutional review board approval [27]. No additional privacy or confidentiality measures were required as the dataset contains no personally identifiable information, with all protected health information having been removed prior to public release.

## Results

### Information Extraction Performance

The details of the findings targeted at information extraction are summarized in Table 1 . The tumor size was correctly identified in 99 (73.3%) of 135 reports. Among the 36 outputs (26.7%) with errors, 23 (17%) lacked size information in their reports, and 22 (16.3%) contained false size information. The remaining errors were attributed to size inaccuracies or empty values despite size information being mentioned in the reports. Tumor location and laterality were accurately identified in 112 (83%) reports. All 23 (17%) reports with errors contained the necessary information but had empty values for laterality, location, or both, with one output indicating an incorrect location. The detailed error analysis is presented in Table 2.

**Table 1.** Summary of lung cancer cases.

Case no.	Side	Lobe	Size (mm)	Lymph node metastasis	Distant metastasis	Data split
Case 1	Left	Upper	18	No	No	Development
Case 2	Right	Lower	12	No	No	Development
Case 3	Left	Upper	28	No	No	Development
Case 4	Left	Upper	40	No	No	Test
Case 5	Left	Upper	48	Yes	No	Test
Case 6	Right	Hilum	Not measurable (due to invasion)	Yes	No	Development
Case 7	Right	Lower	55	Yes	No	Test
Case 8	Left	Upper	Not measurable (due to invasion)	No	No	Test
Case 9	Right	Hilum	43	No	Yes	Development
Case 10	Right	Upper	Not measurable (due to invasion)	No	No	Test
Case 11	Right	Upper	Not measurable (due to invasion)	No	No	Development
Case 12	Right	Lower	Not measurable (due to lung metastasis)	No	Yes	Development
Case 13	Left	Lower	78	Yes	No	Development
Case 14	Left	Upper	85	Yes	No	Test
Case 15	Left	Upper	Not measurable (due to invasion)	Yes	Yes	Test

**Table 2.** Detailed error analysis of tumor size, location, and laterality extraction from radiology reports using large language model (LLM) and rule-based methods.

Category	Extraction methods	
	LLM^a^, n (%)	Rule-based, n (%)
Tumor size (details)		
Correctly identified	99 (73.3)	93 (68.9)
Errors (total)	36 (26.7)	42 (31.1)
Errors (no size information in reports)	23 (17)	0 (0)
False size information generated	22 (16.3)	0 (0)
T classification extracted instead of size	1 (0.7)	0 (0)
Errors (size mentioned in reports)	13 (9.6)	42 (31.1)
Size inaccuracies	8 (5.9)	3 (2.2)
Empty values	5 (3.7)	39 (28.9)
Tumor location and laterality (details)		
Accurately reported	112 (83)	46 (34.1)
Errors (total)	23 (17)	89 (65.9)
Empty values for laterality	9 (6.7)	0 (0)
Empty values for location	5 (3.7)	0 (0)
Empty values for both	8 (5.9)	80 (59.3)
Incorrect location	1 (0.7)	9 (6.7)

a LLM: large language model

The rule-based method correctly identified tumor size in 93 (68.9%) reports, whereas tumor location and laterality were accurately identified in only 46 (34.1%) reports. Among the errors in this method, only 1 case (0.7%) failed to accurately extract size information due to the extraction of multiple sizes. In contrast, for location, the number of errors reached 47 (34.8%) (Figure S3 in Multimedia Appendix 1). Unlike the LLM approach, due to the algorithmic nature of rule-based extraction, there were no cases of false-size information generation. Additionally, as the algorithm extracted laterality and location simultaneously as a single unit, there were no cases where only one of these values was empty; both were either extracted together or left empty.

McNemar’s test showed that the LLM approach was significantly superior to the rule-based method in determining location (*P*<.001) but not size (*P*=.539).

### Clustering Performance

The development data yielded an NMI score of 0.7152, an AMI score of 0.6516, and an FM index of 0.5959, whereas the test data yielded scores of 0.6414 (NMI), 0.5598 (AMI), and 0.5354 (FM).

The proposed method outperformed all previous methods in shared tasks across all evaluation metrics. The detailed results and methods are listed in Table 3. Further details of each method are available in a system paper describing this shared task [28-31].

**Table 3.** Clustering scores on the test data.

Method Description(System ID^a^)	NMI^b^	AMI^c^	FM^d^	Supervised model	LLM^e^
Developed a matrix from word count in radiology reports and applied user-based collaborative filtering for case similarity and clustering, (D1) [28]	0.3569	0.1988	0.2674	No	No
Used paired radiology reports for BERT^f^ input, fine-tuned for same-case identification and clustered based on predictions, (E1) [29]	0.5415	0.1489	0.1814	Yes	No
Generated embeddings from text via multilingual BERT trained on Wikipedia, followed by dimensionality reduction, and K-means clustering, (F1) [30]	0.1744	–0.0117	0.1170	No	No
Labels simplified from the TNM^g^ classification of lung cancer were assigned to each document using BERT-based model for training, and in the test data, these predicted labels were used as groups for clustering, (J1) [31]	0.4622	0.3409	0.3622	Yes	No
This study	0.6414	0.5598	0.5354	No	Yes

a The System IDs are those used in previously shared tasks with the same dataset [5].

b NMI: normalized mutual information

c AMI: adjusted mutual information

d FM: Fowlkes-Mallows index

e LLM: Large language model

f BERT: Bidirectional Encoder Representations from Transformers

g TNM: Tumor, node, metastasis

## Discussion

### Principal Findings

The extraction of lung tumor size showed minimal differences compared to the rule-based method, likely because size information is typically accompanied by standardized units (eg, mm or cm). However, the LLM method significantly outperformed the rule-based method in terms of location extraction, achieving over 80% accuracy and reducing the error rate by half. As demonstrated in Figure S3 in Multimedia Appendix 1, the rule-based method frequently generated multiple incorrect location extractions when reports mentioned various anatomical sites, whereas the LLM method successfully identified the correct tumor location. This finding empirically demonstrates the LLM’s ability to understand and extract information based on context rather than predefined rules. This capability highlights its value for complex information extraction tasks in medical text analysis, where contextual understanding is crucial.

### Comparison to Prior Work

This paper introduces a Japanese LLM algorithm for zero-shot information extraction and clustering that outperforms all previous methods [28-31]. The previous methods (E1, F1, and J1) relied on indirect features extracted by language models, whereas the current approach leverages accurate information extraction through unsupervised learning. The success of this method is particularly notable, given the historically low accuracy of unsupervised methods. By leveraging the LLM’s contextual understanding of information extraction, this study demonstrated the potential for effective clustering of medical reports based on various attributes, including disease severity and lesion localization.

### Strengths and Limitations

This study has several notable strengths including its methodology and implementation. Accurate information extraction and clustering without supervised learning requirements represent a significant advancement in the field. The flexibility of this method through prompt and algorithmic adjustments suggests broad potential applicability, with potential for further performance improvements through prompt optimization [32]. Furthermore, this method shows particular promise for languages with limited training data compared to English, by converting unstructured reports into language-independent structured data, thereby addressing a crucial gap in current medical text analysis.

However, the limitations must be acknowledged. First, validation was limited to small-scale Japanese datasets. While attempts were made to ensure the representativeness of the dataset by including diverse types of lung cancer cases, this limitation constrained the generalizability of the study findings and should be addressed in future studies through multi-institutional validation. Second, the evaluation focused primarily on clustering tasks; which although is a fundamental task in medical text analysis, its performance in other analytical tasks remains unexplored, suggesting the need for a comprehensive evaluation across various applications. Third, while this method shows promise for languages with limited training data, its generalizability to other languages and medical domains requires further investigation.

### Conclusions

The LLM was used to successfully extract important findings from publicly available Japanese radiology reports as highly accurate structured data. By leveraging these structured data, superior results were achieved compared to existing supervised methods for clustering radiology reports. This indicates that employing existing LLMs is effective for solving specific tasks, particularly in languages with a significant shortage of training data compared to English.

## Supplementary material

10.2196/57275 Multimedia Appendix 1 Example of a prompt used as input for the LLM (Japanese original version), pseudo code illustrating the procedure for the rule-based processing, and data representation of extracted information based on the rule-based method.

## Abbreviations

AMI: adjusted mutual information
CLAIM: Checklist for Artificial Intelligence in Medical Imaging
FM: Fowlkes-Mallows index
JSON: JavaScript Object Notation
LLM: large language model
MIMIC-CXR: MIMIC Chest X-ray
NLP: natural language processing
NMI: normalized mutual information
NTCIR-16: NII Testbeds and Community for Information Access Research
