# Supplementary material for: Large Language Model Approach for Zero-Shot Information Extraction and Clustering of Japanese Radiology Reports: Algorithm Development and Validation
Source: JMIR Cancer. 2025 Jan 23;11:e57275. doi: 10.2196/57275 (PMC11867198; doi:10.2196/57275)

Figure S1. Example of a prompt used as input for the LLM (Japanese original version). AAH: Atypical Adenomatous Hyperplasia; AIS: Adenocarcinoma in situ; GGN: Ground Glass Nodule; LLM: large language model; SSN: Subsolid Nodule.


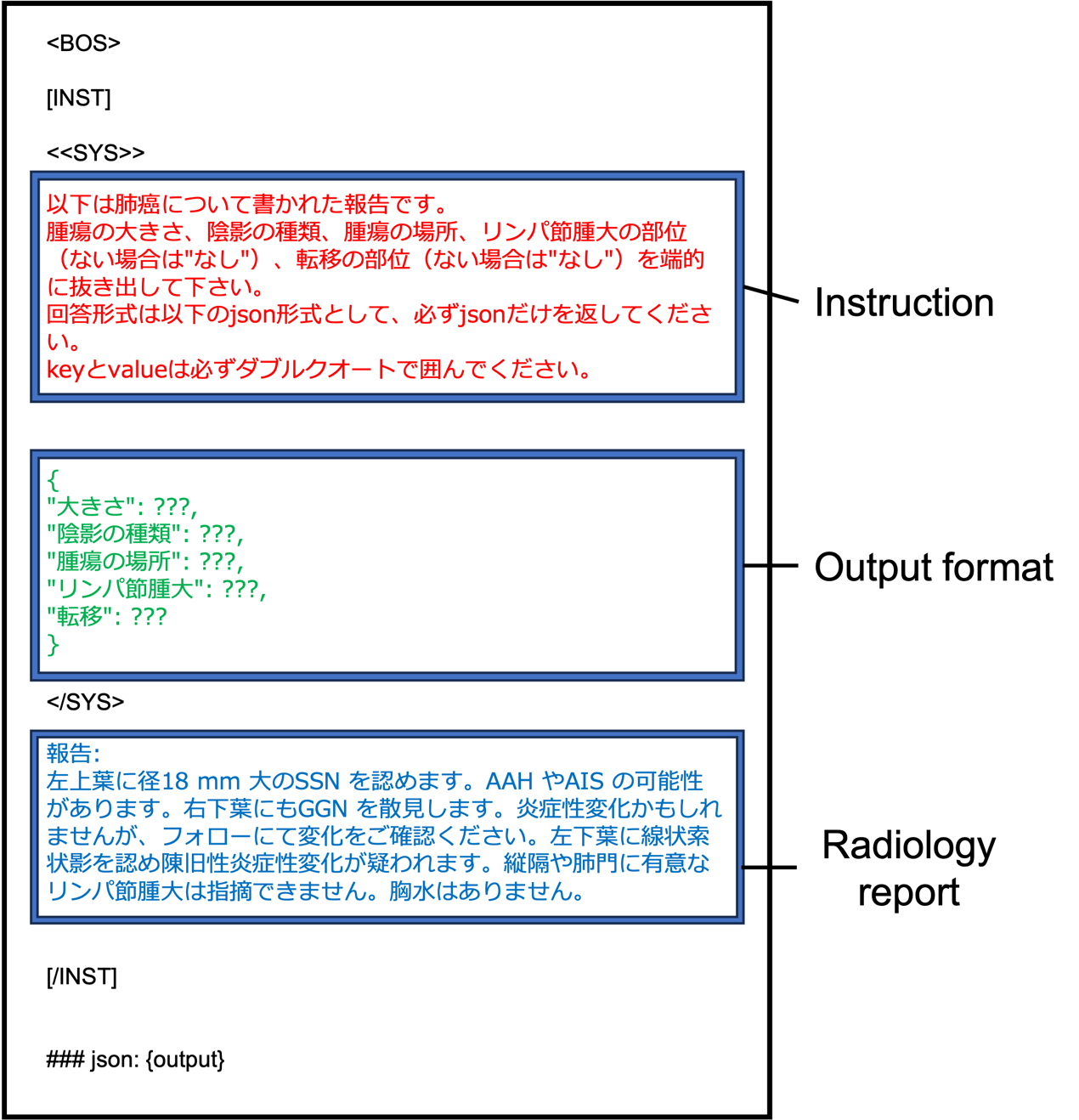


Figure S2. Pseudo code illustrating the procedure for the rule-based processing.


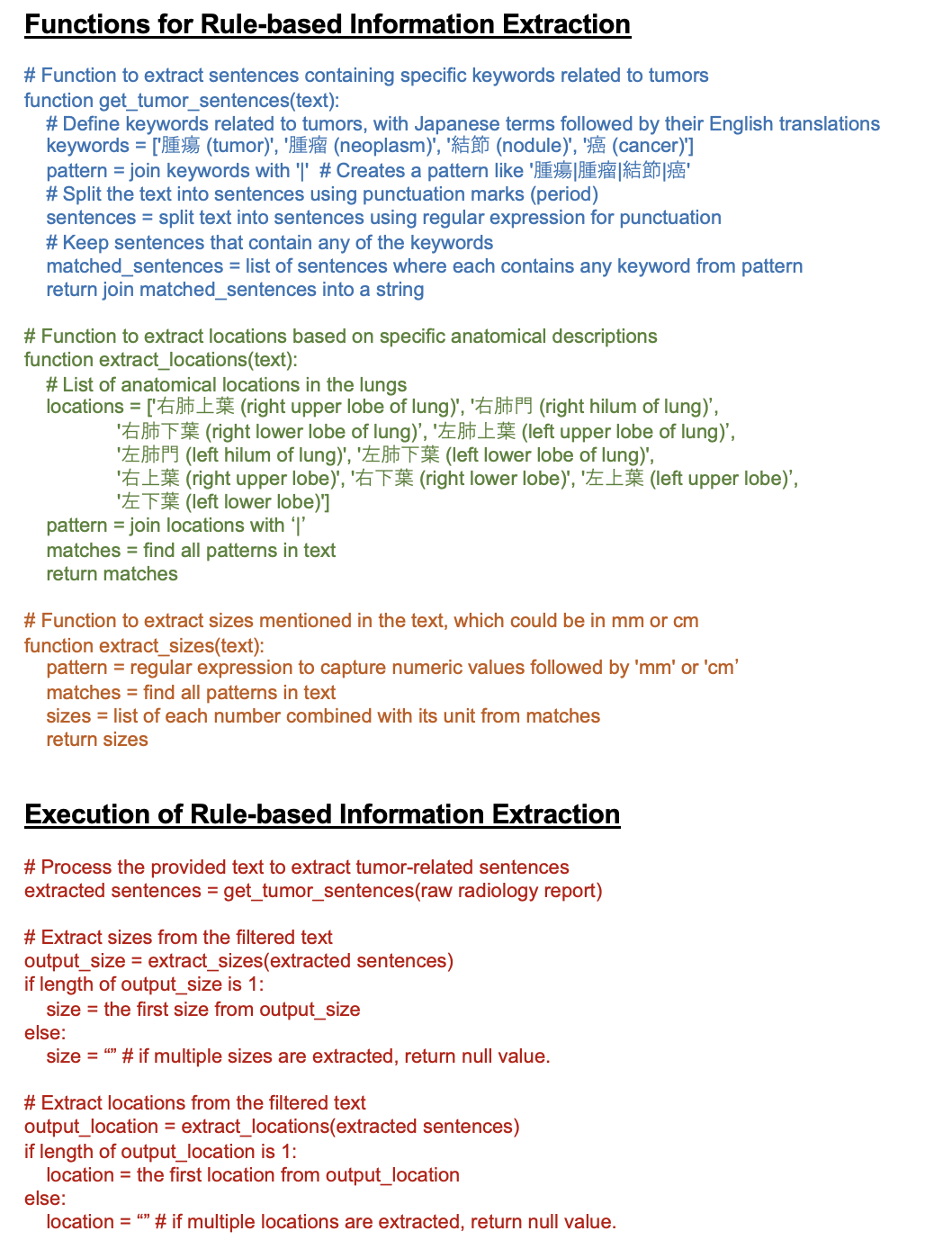


Figure S3. Bar graphs showing the number of extracted information based on the rule-based method.


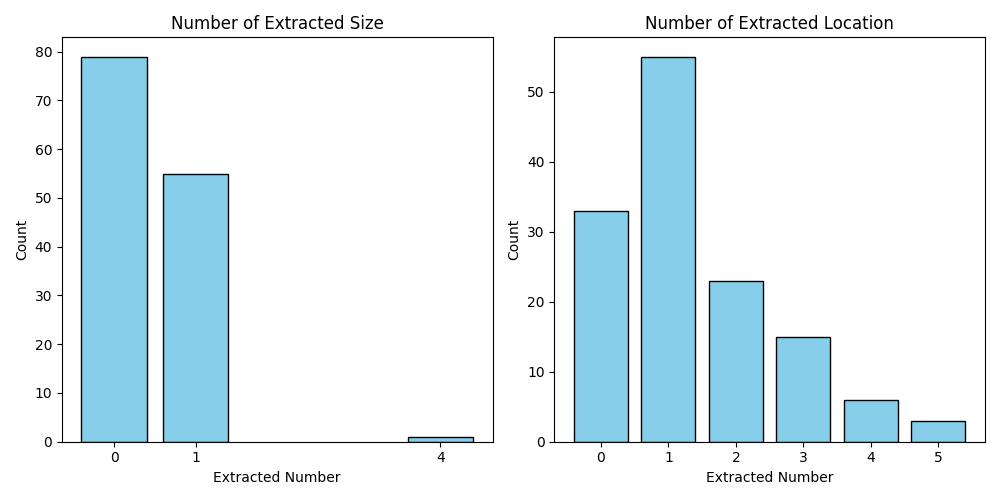

Supplement: Multimedia Appendix 1 [file cancer-v11-e57275-s001.docx]
